# WTX-L/β-arrestin2/LCN2 axis controls vulnerability to ferroptosis in gastric cancer

**DOI:** 10.1016/j.isci.2025.111964

**Published:** 2025-02-07

**Authors:** Yangwei Xu, Xuexia Qian, Guixing Cai, Zhihao Lin, Weiye Huang, Chuangyuan Wang, Hongmei Wu, Yiqiong Zhang, Jingbo Sun, Qingling Zhang

**Affiliations:** 1 Guangdong Cardiovascular Institute, Guangdong Provincial People’s Hospital, Guangdong Academy of Medical Sciences, Guangzhou, Guangdong 510080, China; 2 Department of Pathology, Guangdong Provincial People’s Hospital (Guangdong Academy of Medical Sciences), Southern Medical University, Guangzhou, Guangdong 510080, China; 3 Department of Pathology, Xijing Hospital, Fourth Military Medical University (Air Force Medical University), Xi’an, Shanxi 710032, China; 4 Department of Orthopedic Oncology, Guangdong Provincial People’s Hospital, Southern Medical University, Guangzhou, Guangdong 510080, China; 5 Department of Neurosurgery, Zhujiang Hospital, Southern Medical University, Guangzhou, Guangdong 510282, China; 6 School of Basic Medical Sciences, Southern Medical University, Guangzhou, Guangdong 510282, China

**Keywords:** Molecular biology, Cell biology, Cancer

## Abstract

Gastric cancer (GC) is one of the most prevalent and lethal cancers worldwide. Ferroptosis is a form of iron-dependent regulated cell death emerging as a promising strategy for cancer therapy, whereas the regulation mechanism remains unclear. WTX has been recognized as a potential tumor suppressor, but attempts at targeted therapy have not achieved substantial progress. Further research into the structure, function, and mechanisms is urgently needed. Herein, we identified a long isoform of WTX (WTX-L) as a potent ferroptosis effector in GC. Mechanistically, WTX-L competitively interacts with β-arrestin2, disrupting its direct binding to IκBα and subsequently activating the NF-κB/LCN2 pathway. LCN2 further triggers ferroptosis by significantly increasing the labile Fe^2+^ pool and promoting excessive lipid peroxidation. Blockade of the WTX-L/β-arrestin2/NF-κB/LCN2 axis significantly diminished the activity of ferroptosis inducers (erastin and RSL3) *in vivo*. Collectively, these findings reveal that targeting the ferroptosis vulnerabilities through WTX-L may represent a promising strategy for GC.

## Highlights

• WTX-L, rather than WTX-S, serves as a positive regulator of ferroptosis
• WTX-L facilitates iron-dependent oxidative damage during ferroptosis via LCN2
• WTX-L interacts with β-arrestin2 to activate the NF-κB signaling pathway
• Targeting the WTX-L/β-arrestin2/LCN2 axis is a promising therapeutic strategy for GC

## Graphical abstract

**Figure:**
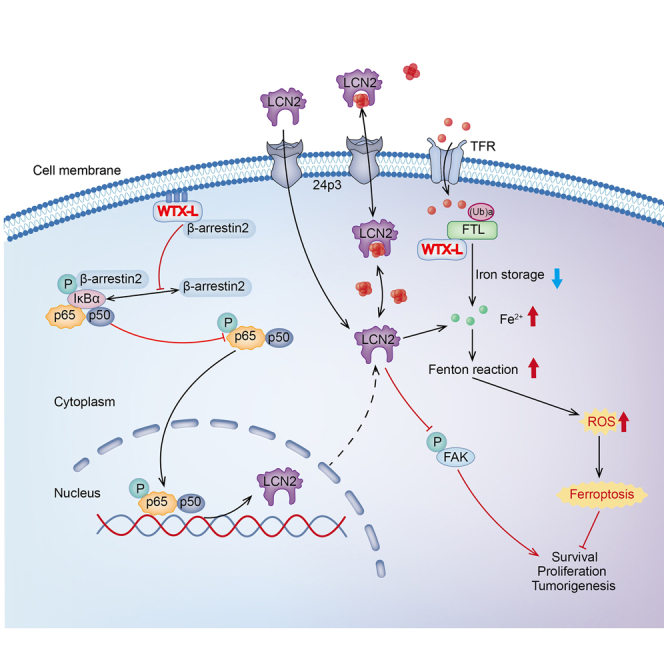

## Introduction

Gastric cancer (GC) is the fifth most common cancer and the third leading cause of cancer-related deaths worldwide.^1^^,^^2^ At present, remarkable progress has been achieved for GC treatment such as surgery, chemotherapy, and targeted therapies (including trastuzumab, ramucirumab, and nivolumab or pembrolizumab), but the 5-year overall survival rates for patients with GC are still unsatisfactory, which is associated with its postoperative recurrence, chemoresistance, and metastasis.^1^^,^^3^^,^^4^ Herein, finding effective biomarkers and/or targets is imperative to explore the mechanisms underlying GC progression for developing more efficient therapeutic strategies.

Ferroptosis is a regulated cell death that depends on the content of iron and lipid hydroperoxide. Large amounts of lipids accumulate in the cells, disrupting the balance of the redox reaction and ultimately leading to cell death, which is closely related to iron homeostasis, lipid metabolism, and amino acid metabolism.^5^ Given that ferroptosis is the result of iron-modulated oxidative damage, tumor cells have a higher propensity for ferroptosis than normal cells because of their increased metabolic activity and reactive oxygen species load, which means that they usually require higher levels of iron and amino acids to enable their growth and proliferation.^6^^,^^7^ Currently, the activation of ferroptosis by several small molecules and Food and Drug Administration-approved clinical drugs in cancer cells, and the efficacy of tumor suppression by ferroptosis inducers (such as erastin and RSL3) in various experimental cancer models, underlines the potential of ferroptosis as a novel anticancer therapy.^7^^,^^8^ Therefore, it is urgent to investigate the diverse vulnerabilities of cancer cells to ferroptosis and explore mechanisms and strategies for targeting ferroptosis in cancer.

Tumor suppression is one of the most important inherent physiological functions of ferroptosis. Ferroptosis significantly enhances the tumor-suppressive effect of some tumor suppressor genes, such as BAP1 and p53. The tumor-suppressor activity of BAP1 is partly mediated by ferroptosis through deubiquitination of H2A on the SLC7A11 promoter, resulting in repression of SLC7A11 expression.^9^ In addition to being involved in canonical functions such as cell-cycle arrest, senescence, or apoptosis,^10^ P53 is also involved in tumor suppression by regulating ferroptosis through controlling metabolism and redox state.^11^ Thus, their antitumor activity is even more pronounced than that of non-ferroptosis-related tumor suppressors. Unlike other tumor suppressor genes, APC membrane recruitment 1 (AMER1)/FAM123B/Wilms tumor gene on the X chromosome (WTX) gene is inactivated by a monoallelic “single-hit” event.^12^ And the protein name of WTX (UniProt ID, Q5JTC6) is APC membrane recruitment protein 1 or Wilms tumor gene on the X chromosome protein. Among its known mechanisms of functions, WTX not only negatively regulates WNT/β-catenin signaling by promoting β-catenin ubiquitination and degradation^13^ but also inhibits NRF2 protein degradation through competitive binding to KEAP1.^14^ Since aberrant activation of the Wnt/β-catenin signaling confers ferroptosis resistance^15^ and NRF2 controls iron homeostasis and ferroptosis through HERC2 and VAMP8,^16^ this gives us a hint that WTX, as their upstream regulatory molecule, may exhibit tumor-suppressor activity by modulating ferroptosis. In our previous study, WTX expression is positively correlated with the overall survival of patients with GC and restoring WTX significantly inhibits the proliferation, migration, and invasion ability of GC cells.^17^^,^^18^^,^^19^ Collectively, these studies demonstrated that WTX may play critical roles in GC tumorigenesis and the regulatory mechanisms of WTX-mediated ferroptosis in tumor suppression and biology still need further exploration. In addition, WTX has been identified in two splice variants: the full-length form (WTX-L) and a variant lacking a significant portion of the membrane association domain (WTX-S). However, the specific roles of the WTX isoforms (WTX-S and WTX-L) in general disease and specifically in GC ferroptosis remain poorly understood.

In this study, we demonstrate that WTX plays a crucial role in the ferroptosis of GC through a series of cellular, molecular, and pharmacological analyses. Mechanistically, we found that unspliced WTX-L localizes to the plasma membrane and forms a complex with β-arrestin2. This interaction disrupts the direct binding of β-arrestin2 to IκBα, leading to the upregulation of LCN2 expression, which is essential for generating sufficient lipid reactive oxygen species (ROS) to initiate ferroptosis. These findings will benefit the potential development of therapeutic strategies involving the induction of ferroptosis in GC.

## Results

### WTX-L, instead of WTX-S, acts as a positive ferroptosis regulator

**Figure 1:**
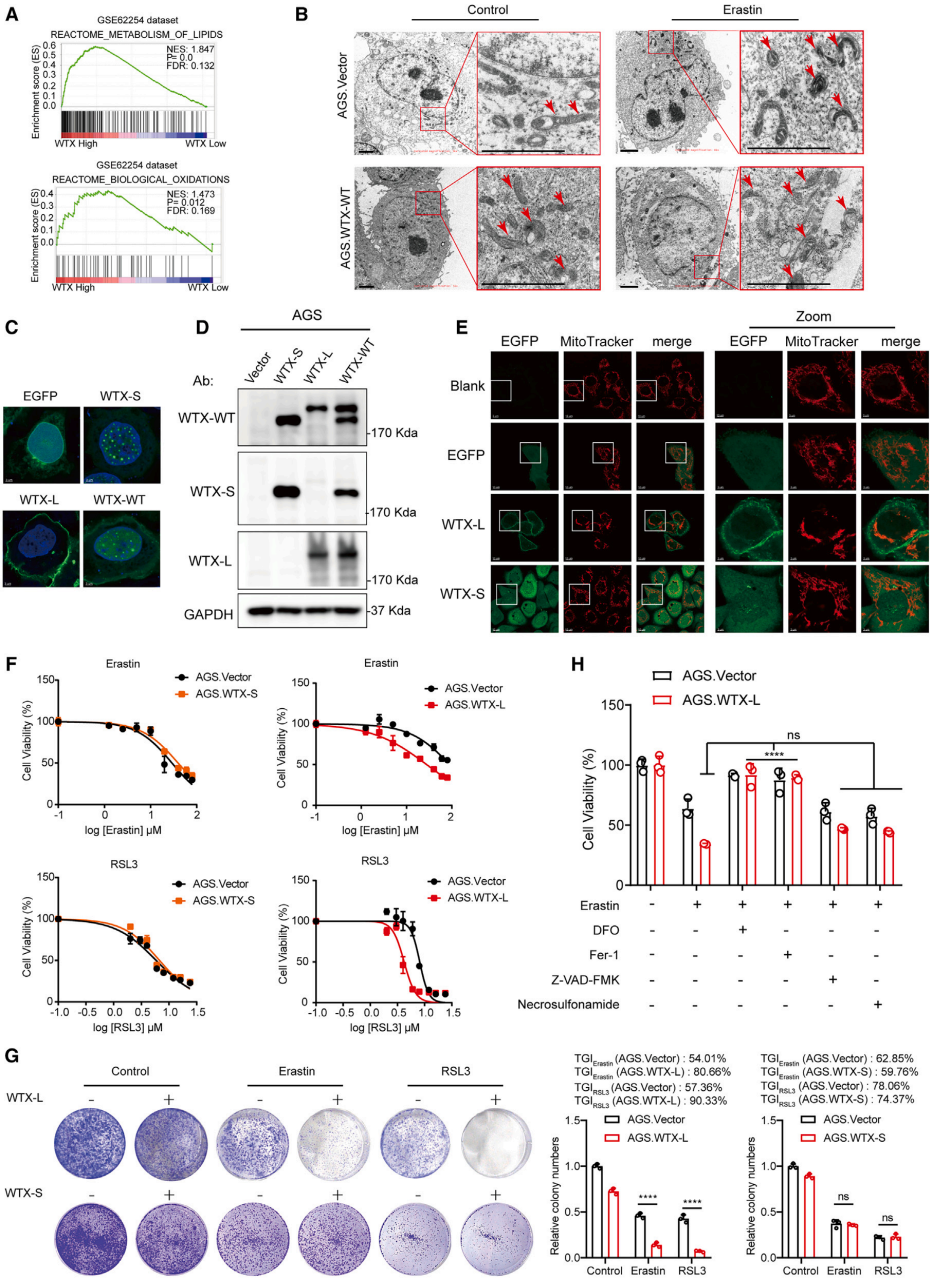
WTX acts as a ferroptosis regulator (A) Gene set enrichment analysis demonstrated lipid metabolism and biological oxidation pathways in the GC group with high WTX expression from the GEO: GSE62254 dataset. (B) The morphological changes of mitochondria in AGS.Vector and AGS.WTX-WT cells were detected by transmission electron microscopy in the absence or presence of 3 μM erastin. Lower scale bars, 1 μm. (C) Confocal-fluorescence microscopy images showed intracellular localization detection of EGFP, WTX-L, WTX-S, and WTX-WT in AGS cells. Scale bars, 3 μm. (D) Protein expression of EGFP-tagged WTX-WT, WTX-L, and WTX-S in AGS cells was examined by western blot assay. (E) Confocal-fluorescence microscopy showed mitochondrial morphology in AGS.blank AGS.Vector, AGS.WTX-L, and AGS.WTX-S cells with MitoTracker probe. Scale bars, 10 μm; scale bar (zoom), 3 μm. (F) AGS.Vector, AGS.WTX-L, and AGS.WTX-S cells were exposed to indicated concentrations of erastin (top) and RSL3 (bottom) for 24h. Cell viability was measured by CCK8 assay. (G) Colony formation assay analyzed the cell survival of AGS.Vector, AGS.WTX-L, and AGS.WTX-S cells exposed to erastin (1μM) and RSL3 (0.5μM). TGI, tumor growth inhibition rate. (H) AGS.Vector and AGS.WTX-L cells were treated with DMSO or 3μM erastin for 6h in the presence or absence of several small-molecular inhibitors (100 μM DFO, 1 μM ferrostatin-1, 0.5 μM necrosulfonamide, 5 μM Z-VAD-FMK). The cell viability was detected by CCK8 assay. Data are represented as mean ± SEM (n = 3 per group, unless otherwise indicated). ∗∗∗∗*p* < 0.0001, ns: no significance.

In our previous studies, GC cells have been chosen for loss- or gain-of-function studies based on their low or high endogenous WTX expression, respectively.^19^ To further investigate the role of WTX in GC progression, we performed gene set enrichment analysis (GSEA) in GEO: GSE62254 containing 300 GC specimens and found significant enrichment of pathways related to lipid metabolism and biological oxidation in tumors with high WTX expression (Figure 1A). DCF staining further revealed a significant increase in the fluorescence intensity of DCF in AGS.WTX-WT cells, indicating that WTX overexpression promotes the formation of ROS (Figures S1A and S1B). Given the close association among lipid metabolism, biological oxidation, and ferroptosis, we hypothesized whether alteration of WTX expression might cause ferroptosis in GC. Erastin and RSL3, the two major compounds regularly used to induce ferroptosis, are considered as “classical” inducers of this regulated cell death subroutine.^8^^,^^20^ To determine our hypothesis, we measured cell morphology change and cell viability of AGS.Vector/AGS.WTX-WT and MKN45.shNC/MKN45.shWTX cells after erastin and RSL3 treatment. Compared with the control cells, WTX overexpression cells treated with erastin and RSL3 exhibited a notable increase in the number of cells undergoing the “ballooning” morphology indicative of ferroptotic cell death (Figure S1C). At the same time, WTX overexpression markedly increased erastin-induced and RSL3-induced growth inhibition, whereas WTX knockdown exhibited the opposite effect (Figures S1D–S1F). Morphological change of mitochondria is one of the critical features of ferroptosis.^21^ Thus, we used transmission electronic microscopy (TEM) to observe the mitochondria morphological changes in AGS.WTX-WT cells, including mitochondrial fragmentation, vacuolization, and cristae enlargement, which were further aggravated under erastin treatment (Figure 1B). Overall, these results showed that WTX enhances the sensitivity of GC cells to ferroptosis.

It has been reported that two endogenous WTX isoforms, namely, WTX-S and WTX-L, are present in eukaryotic cells due to alternative splicing.^22^ To explore which isoform accounts for ferroptosis, we generated EGFP-tagged constructs for three WTX transcripts (WTX-WT, WTX-L, and WTX-S) and examined their subcellular localization. The results showed that WTX-L was primarily located in the plasma membrane and cytoplasm, WTX-S accumulated as puncta in the nucleus, while WTX-WT exhibited a mixed pattern with expression in the plasma membrane, cytoplasm, and nucleus (Figures 1C and 1D). A comparison was made between blank cells and control cells with a network of elongated mitochondria, and cells with WTX-L overexpression showed mitochondrial fragmentation accumulation, while WTX-S did not (Figure 1E), indicating that WTX-L destroyed mitochondrial homeostasis. Consistently, cell viability (Figure 1F) and colony formation assay (Figure 1G) both revealed that overexpression of WTX-L, instead of WTX-S, markedly promoted cytotoxicity of erastin and RSL3 to AGS cells. Furthermore, as shown in Figure 1H, the effect of WTX-L overexpression could be completely reversed by ferroptosis inhibitors (e.g., deferoxamine [DFO] and Fer-1) but not by apoptosis inhibitor (e.g., Z-VAD-FMK) and necroptosis inhibitor (e.g., necrosulfonamide). Collectively, these results demonstrated that WTX-L is a positive regulator of ferroptosis.

Accumulation of lipid peroxidation is a major feature of ferroptosis, typically mitigated by the antioxidant enzyme GPX4. Thus, GPX4 can be considered a marker of ferroptosis.^23^ Accordingly, we further evaluated the differences in GPX4 expression between WTX-L-overexpressing cells and control cells under erastin treatment. The results showed that erastin treatment significantly reduced GPX4 expression in WTX-L-overexpressing cells compared with control cells (Figure S1G). Specifically, WTX-L retains the internal splice donor site without altering the amino acid sequence, whereas WTX-S features in-frame deletions of residues 50–326. Thus, we constructed an expression vector WTX-1-326aa to determine its role in ferroptosis. Additionally, the cell viability assay showed that WTX-1-326aa had a lower impact on enhancing ferroptosis sensitivity compared with WTX-L (Figure S1H), suggesting that residues 50–326 of WTX are crucial for the biological function of ferroptosis, and that WTX-L is also involved in the ferroptosis process, possibly aided by its other functional domains.

### WTX-L promotes iron-dependent oxidative damage in ferroptosis

**Figure 2:**
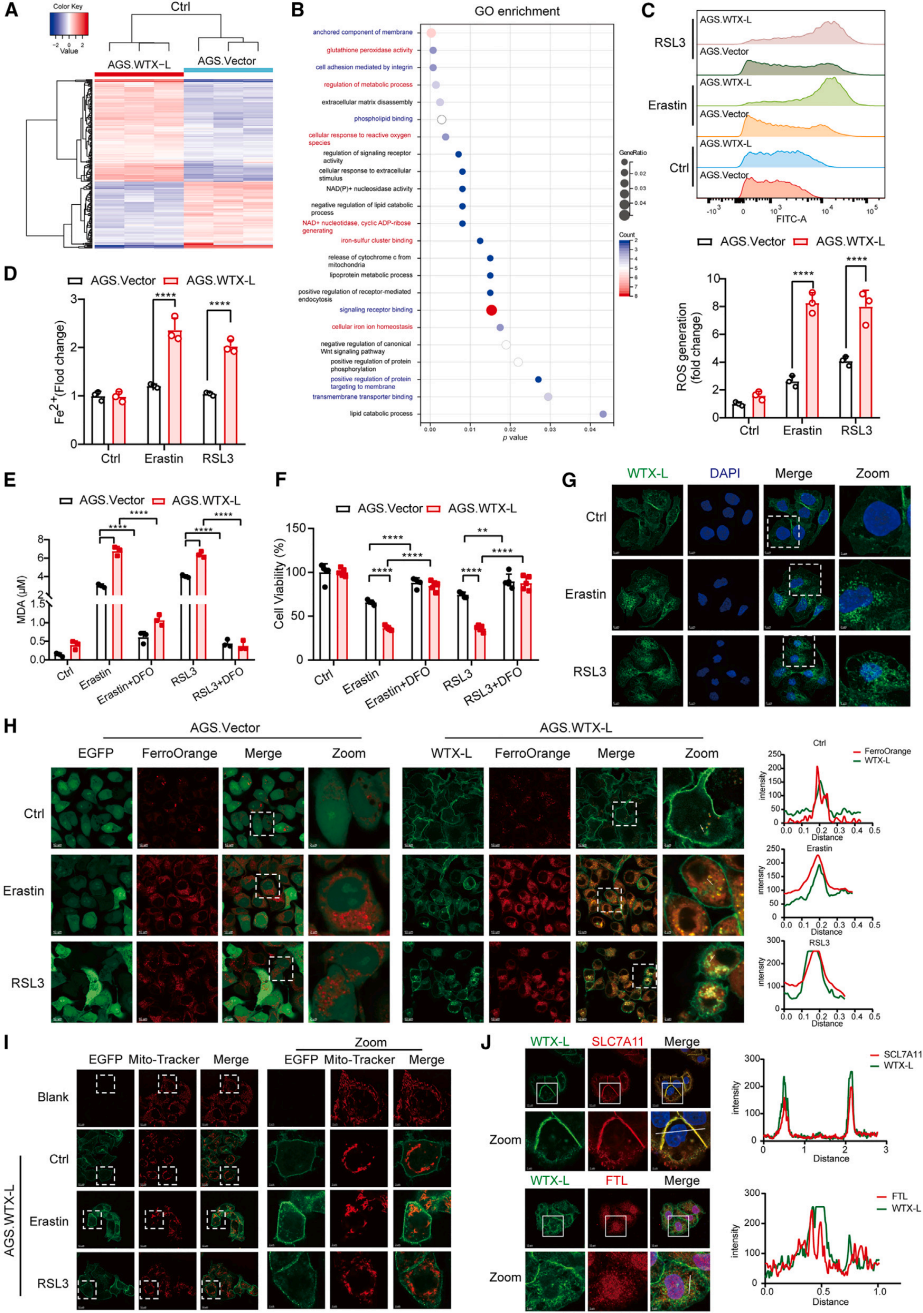
WTX-L promotes iron-dependent oxidative damage in ferroptosis (A and B) Hierarchical clustering analysis (A) and GO analysis (B) of differentially expressed genes between AGS.Vector and AGS.WTX-L cells (fold change>2.0). (C and D) AGS.Vector and AGS.WTX-L cells were exposed to 3μM erastin and RSL3 for 8h. Intracellular ROS levels (C) and Fe^2+^ levels (D) in WTX-L cells compared with Vector cells were detected through flow cytometry assay and iron detection assay, respectively. (E) The MDA assay detected lipid peroxidation in AGS.Vector and AGS.WTX-L cells pretreated with indicated concentrations of erastin and RSL3. (F) CCK8 assay analyzed the cell viability of AGS.Vector and AGS.WTX-L cells exposed to indicated concentrations of erastin and RSL3. (G) Immunofluorescence assay was performed to determine the effect of erastin and RSL3 on the subcellular localization of WTX-L. Scale bars, 5 μm; scale bar (zoom), 2 μm. (H) Confocal images of AGS.Vector and AGS.WTX-L cells labeled with FerroOrange after treatment with 3μM erastin or 1μM RSL3 for 24h. Red and green colors indicate Fe^2+^ and WTX-L, respectively. Scale bars, 10 μm; scale bar (zoom), 2 μm. (I) Confocal images showed the mitochondria morphology in AGS.Vector and AGS.WTX-L cells stained with MitoTracker. Red and green colors indicate mitochondria and WTX-L, respectively. Scale bars, 10 μm; scale bar (zoom), 3 μm. (J) Immunofluorescence staining for WTX-L and endogenous SLC7A11 or FTL in AGS.WTX-L cells. The right panel shows the colocalization results. Scale bars, 3 μm. Data are represented as mean ± SEM (n = 3 per group, unless otherwise indicated). ∗∗*p* < 0.01, ∗∗∗∗*p* < 0.0001.

To explore the mechanisms underlying WTX-L-mediated ferroptosis, RNA sequencing (RNA-seq) was performed, and 200 differentially expressed genes (DEGs) (121 upregulated and 79 downregulated genes) were identified between AGS.WTX-L and AGS.Vector cells (adjust *p* < 0.05, |Log2FC|>1) (Figure 2A). The biological function of the DEGs induced by WTX-L overexpression was next investigated by Gene Ontology (GO) enrichment analysis. The results indicated that DEGs were significantly enriched in multiple ferroptosis-related terms, such as glutathione peroxidase activity, cellular iron ion homeostasis, and lipid catabolic process (Figure 2B). Interestingly, we also performed RNA-seq in AGS.WTX-S cells (Figure S2A) and GO enrichment analysis revealed completely different results compared with AGS.WTX-L, with no signaling pathways related to ferroptosis (Table S1), which further confirmed the critical role of WTX-L in regulating ferroptosis. Based on the transcriptome results, we detected lipid ROS generation and intracellular Fe^2+^ levels in AGS.Vector/AGS.WTX-L (Figures 2C and 2D) and MKN45.shNC/MKN45.shW-L (Figures S2B and S2C) cells after erastin and RSL3 treatment. The results showed that WTX-L expression positively correlated with erastin-induced or RSL3-induced lipid ROS formation and Fe^2+^ accumulation. The increased oxidative stress that resulted from iron overload may cause ferroptosis via targeting membrane lipids. Thus, we further found that overexpression of WTX-L in AGS cells promoted erastin-induced or RSL3-induced lipid peroxidation as measured by quantifying malondialdehyde (MDA) levels (Figure 2E). The marked reversal of this effect could be achieved by DFO (an iron chelator). Consistent results were obtained through cell viability assay (Figure 2F).

Of note, when we observed AGS.WTX-L cells through CFM (Confocal microscope), we accidentally found that WTX-L tended to accumulate and assemble as spots in cells upon erastin and RSL3 treatment (Figure 2G). To determine whether this phenomenon was associated with Fe^2+^ accumulation during ferroptosis, we conducted immunofluorescence (IF) assay to simultaneously detect WTX-L and chelatable iron by using an Fe^2+^ iron probe known as FerroOrange. As shown in Figure 2H, there was significant colocalization between WTX-L and FerroOrange in AGS.WTX-L cells following erastin and RSL3 treatment, indicating a potential interaction between WTX-L and the free iron pool. We further detected mitochondria morphology in AGS.WTX-L cells through MitoTracker staining and found that WTX-L markedly increased mitochondrial fragmentation levels around the nucleus upon erastin and RSL3 treatment (Figure 2I), which were consistent with the above findings. Besides, contrary results were found in MKN45.shW-L cells (Figure S2D). Based on recent study reporting that exogenous WTX interacts with SLC7A11 or FTL in HEK293T cells,^24^ we found that isoform WTX-L was colocalized with endogenous SLC7A11 and FTL in AGS.WTX-L cells, further indicating that WTX-L modulates iron homeostasis. Taken together, these results demonstrated that WTX-L led to a significantly increased pool of labile free iron, which triggers ROS overload and induces ferroptosis.

### LCN2 acts as an effector gene in WTX-L-induced ferroptosis

**Figure 3:**
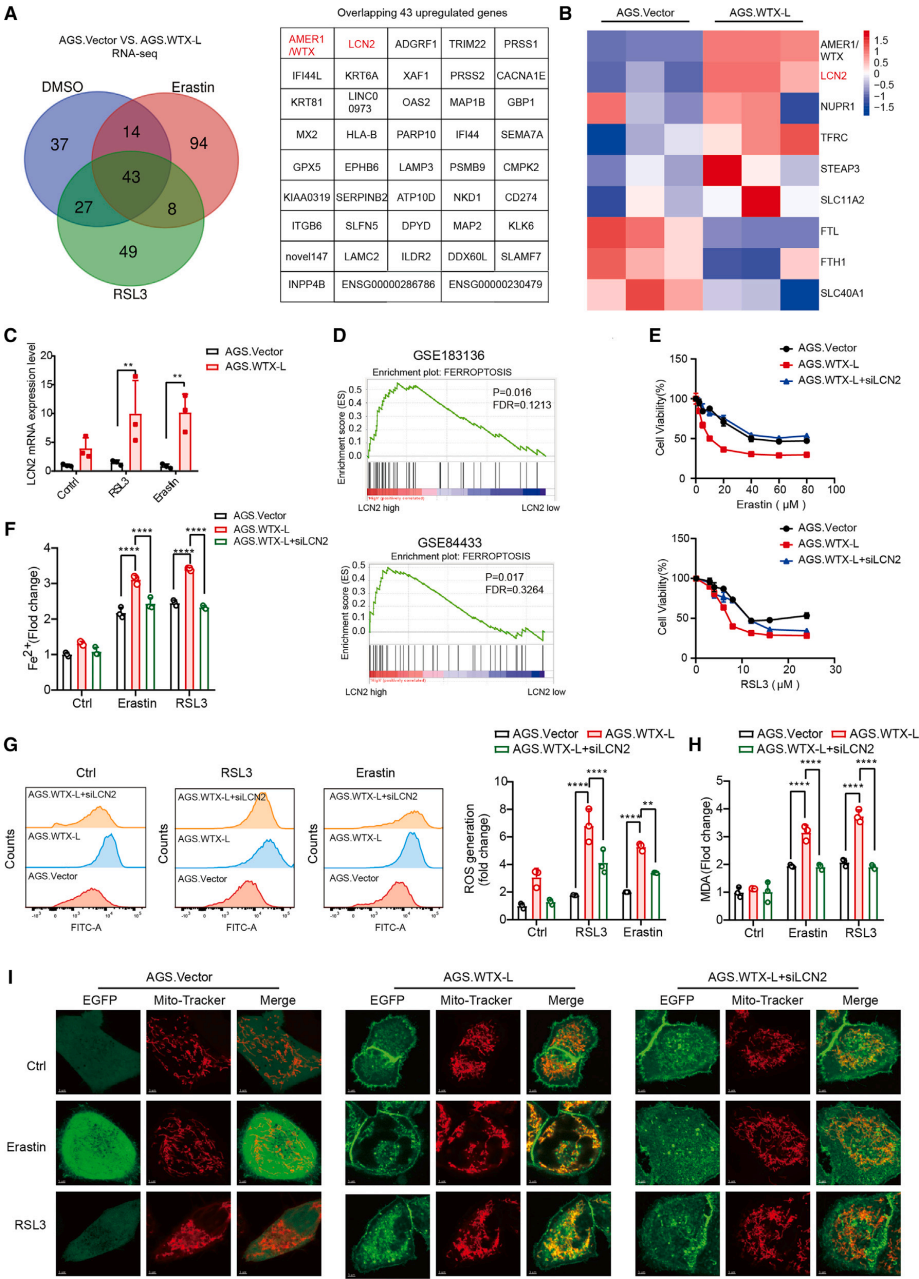
LCN2 acts as an effector gene in WTX-L-mediated ferroptosis sensitivity (A) The Veen map displayed the number of genes that were significantly differentially expressed in AGS.Vector/WTX-L cells treated with erastin or RSL3 versus AGS.Vector/WTX-L cells treated with DMSO (more than 1-fold and *p* < 0.05). (B) Heatmap of RNA-seq data from AGS.Vector and AGS.WTX-L cells representing significant differentially expressed genes (iron-metabolism-associated genes). Blue bar indicates downregulated genes. Red bar indicates upregulated genes. (C) RT-qPCR showed the LCN2 expression levels in AGS.Vector and AGS.WTX-L cells following treatment with 3μM erastin or 1μM RSL3. (D) GSEA demonstrated the ferroptosis pathway in the GC group with high LCN2 expression from GEO: GSE183136 and GEO: GSE84433 datasets. (E) AGS cells of the indicated groups were exposed to multiple concentrations of erastin (top) and RSL3 (bottom) for 24 h. The cell viability was determined by CCK8 assays. (F) AGS cells of the indicated groups were treated with 3μM erastin or 1μM RSL3 for 24h. Relative intracellular Fe^2+^ levels were measured by iron detection assay. (G) AGS cells of the indicated groups were treated with 3μM erastin or 1μM RSL3 for 24h. Intracellular ROS levels were detected through flow cytometry assay. (H) AGS cells of the indicated groups were treated with 3μM erastin or 1μM RSL3 for 24h. Lipid peroxidation was measured by MDA assay. (I) AGS cells of the indicated groups were treated with 3μM erastin or 1μM RSL3 for 24h. Mitochondria morphology was shown after staining with MitoTracker. Red and green colors indicate mitochondria and EGFP, respectively. Scale bars, 3 μm. Data are represented as mean ± SEM (n = 3 per group, unless otherwise indicated). ∗∗*p* < 0.01, ∗∗∗∗*p* < 0.0001.

Given the critical roles of WTX-L in cellular iron ion homeostasis, we seek to precisely elucidate the underlying mechanism through transcriptome analysis. We further conducted RNA-seq in AGS.WTX-L/AGS.Vector cells following erastin and RSL3 treatments. As shown in Figure S3A, overexpression of WTX-L resulted in 268 (159 upregulated and 109 downregulated genes) and 205 (127 upregulated and 78 downregulated genes) DEGs upon erastin and RSL3 treatments, respectively. A total of 43 overlapping upregulated genes were identified in all three groups of DEGs (Figure 3A). It is known to all that intracellular iron level is dependent on its uptake, storage, and release.^25^^,^^26^ Briefly, Fe^3+^ is absorbed by TFRC, reduced to Fe^2+^ by STEAP3, and then released from endosome to cytoplasm by SLC11A2. FTH1 and FTL are both major ferritins that function as iron storage proteins. As for release, it requires the participation of iron transporters such as SLC40A1 and LCN2. Among the vital genes for iron metabolism mentioned above, LCN2 was the only identified overlapping gene through RNA-seq (Figure 3B), and RT-qPCR results showed that the expression of other genes in WTX-L-overexpressing cells was not significantly different from that in control cells (Figure S3B). Consistent with the RNA-seq data, WTX-L markedly enhanced erastin-induced and RSL3-induced mRNA expression of LCN2 (Figure 3C). Previous researches have shown that LCN2 acts in distinct roles in regulating ferroptosis of distinct cancers, and its role in GC ferroptosis remains unclear. GSEA results, obtained by analyzing the expression profile data of patients with GC from the GEO: GSE183136 and GEO: GSE84433 datasets both indicated that the ferroptosis pathway was markedly enriched in GC samples harboring high LCN2 expression (Figure 3D). These results suggested that LCN2 may play a catalytic role in WTX-L-mediated GC ferroptosis.

Subsequently, we performed rescue experiments using LCN2 siRNA and overexpressed plasmid. The expression efficiency of LCN2 was confirmed by RT-qPCR (Figure S3C) and western blot assays (Figures S3D–S3F), respectively. Cell viability assay revealed that silencing LCN2 significantly attenuated the role of WTX-L in promoting erastin and RSL3-induced cell death (Figure 3E). Meanwhile, intracellular Fe^2+^ (Figure 3F), ROS generation (Figure 3G), MDA levels (Figure 3H), and mitochondria morphology also partially restored upon LCN2 knockdown (Figure 3I). On the contrary, overexpressing LCN2 markedly rescued the cell death and ROS levels inhibited by WTX-L knockdown (Figures S3G and S3H). Collectively, these results demonstrated that LCN2 was the key effector gene in WTX-L-induced ferroptosis. However, the mechanisms underlying WTX-L in upregulating LCN2 remain to be further elucidated.

### WTX-L interacts with β-arrestin2 to activate the NF-κB pathway and upregulate LCN2 expression

**Figure 4:**
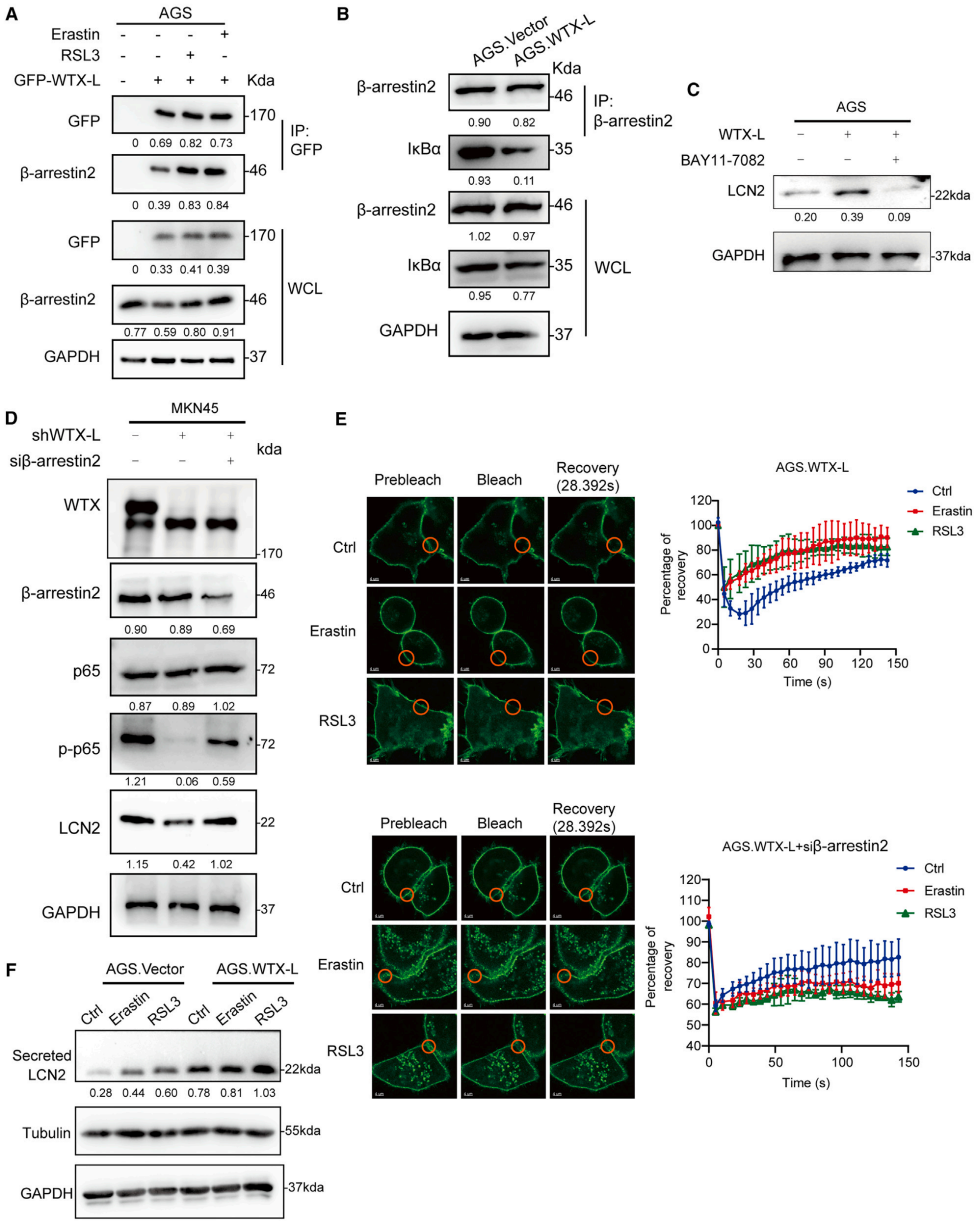
The interaction between WTX-L and β-arrestin2 promotes LCN2-mediated ferroptosis via the NF-κB pathway (A) CoIP analysis of the interactions between WTX-L and β-arrestin2 in AGS cells following treatment with 3μM erastin or 1μM RSL3 for 24h. (B) CoIP analysis of the interactions between IκBα and β-arrestin2 in AGS.Vector and AGS.WTX-L cells. (C) Western blot analysis of LCN2 expression levels in AGS.Vector and AGS.WTX-L cells with or without BAY-7082 treatment. (D) Western blot analysis of indicated protein levels in MKN45.shNC and MKN45.shWTX-L cells with or without BAY11-7082 treatment. (E) FRAP recovery experiment of EGFP-WTX-L upon erastin and RSL3 treatment in AGS.WTX-L cells with or without β-arrestin2 knockdown. The solid circle shows the photobleaching region. Scale bars, 4 μm. (F) Western blot showed the content of secreted LCN2 in AGS.Vector and AGS.WTX-L cells following treatment with 3μM erastin or 1μM RSL3. Data are represented as mean ± SEM (n = 3 per group, unless otherwise indicated).

Previous researches have reported that LCN2 could be transcriptionally upregulated by NF-κB in liver cancer cells and that, in NB4 cells,^27^ membrane WTX is capable of interacting with β-arrestin2, which has been recognized to be a negative regulator of the NF-κB pathway.^28^^,^^29^ Thus, we hypothesized that WTX-L (membrane localization) might upregulate LCN2 via the β-arrestin2/NF-κB axis. Coimmunoprecipitation (coIP) assay was first performed in AGS cells, and the results confirmed the interaction between WTX-L and β-arrestin2, which could be promoted by erastin and RSL3 treatments (Figure 4A). Notably, further coIP assay indicated that WTX-L competitively inhibited the interaction between β-arrestin2 and IκBα, a critical member of the NF-κB inhibitor family, which might result in activation of the downstream NF-κB pathway and target genes (Figure 4B). Subsequent western blot assay confirmed our hypothesis. Rescue experiments were conducted using BAY11-7082 (an inhibitor for NF-κB pathway) and β-arrestin2 siRNA. The results showed that the WTX-L-mediated LCN2 upregulation could be significantly reverted by BAY11-7082 treatment (Figure 4C). To determine whether WTX-L-mediated activation of the NF-κB pathway is essential for RSL3- and erastin-induced ferroptosis, we inhibited the NF-κB pathway using BAY11-7082 and evaluated its effect on sensitivity to ferroptosis. The results demonstrated that inhibition of the NF-κB pathway reduced WTX-L-induced ferroptosis (Figure S4A). Conversely, silencing β-arrestin2 activated the NF-κB signaling pathway and restored LCN2 expression inhibited by WTX-L knockdown (Figure 4D). Taken together, these results demonstrated the vital roles of the WTX-L/β-arrestin2/NF-κB axis in upregulating LCN2 and inducing ferroptosis.

We also delved into the interaction details of WTX-L and β-arrestin2 on cytomembrane from the perspective of protein fluidity through FRAP (fluorescence recovery after photobleaching) assay. As shown in Figure 4E, erastin and RSL3 treatments markedly decreased the immobilized WTX-L fraction in the plasma membrane, whereas this effect could be completely reverted by silencing β-arrestin2. The results of FRAP showed that β-arrestin2 was required for WTX-L membrane stabilization. It was also indirectly implied that erastin and RSL3 promote membrane binding of WTX-L and β-arrestin2. Moreover, cell-secreted LCN2 enters the cell through its interaction with the LCN2 receptor and plays a key role in intracellular iron homeostasis. Therefore, we examined whether WTX-L overexpression affects extracellular secretion of LCN2 during ferroptosis by western blot. The result showed that WTX-L promotes erastin and RSL3-induced LCN2 secretion (Figure 4F), suggesting that WTX-L-mediated extracellular release of LCN2 may trigger ferroptosis in neighboring cells.

In addition, KEGG (Kyoto Encyclopedia of Genes and Genomes) analysis in AGS.WTX-L/AGS.Vector cells following erastin and RSL3 treatments consistently revealed a significant enrichment in focal adhesion pathway, which is tightly associated with membrane viscoelasticity (Figure S4B). Combined with previous reports, LCN2 blocked cell adhesion and invasiveness through the inhibition of the focal adhesion kinase (FAK) phosphorylation.^30^ Both the IF assay and western blot assay unveiled that WTX-L suppression inhibited the expression of P-FAK (Figures S4C and S4D), demonstrating that WTX-L may inhibit focal adhesion by upregulating LCN2 during ferroptosis.

### Inhibition of WTX-L impairs sensitivity of GC cells to erastin and RSL3 *in vivo*

**Figure 5:**
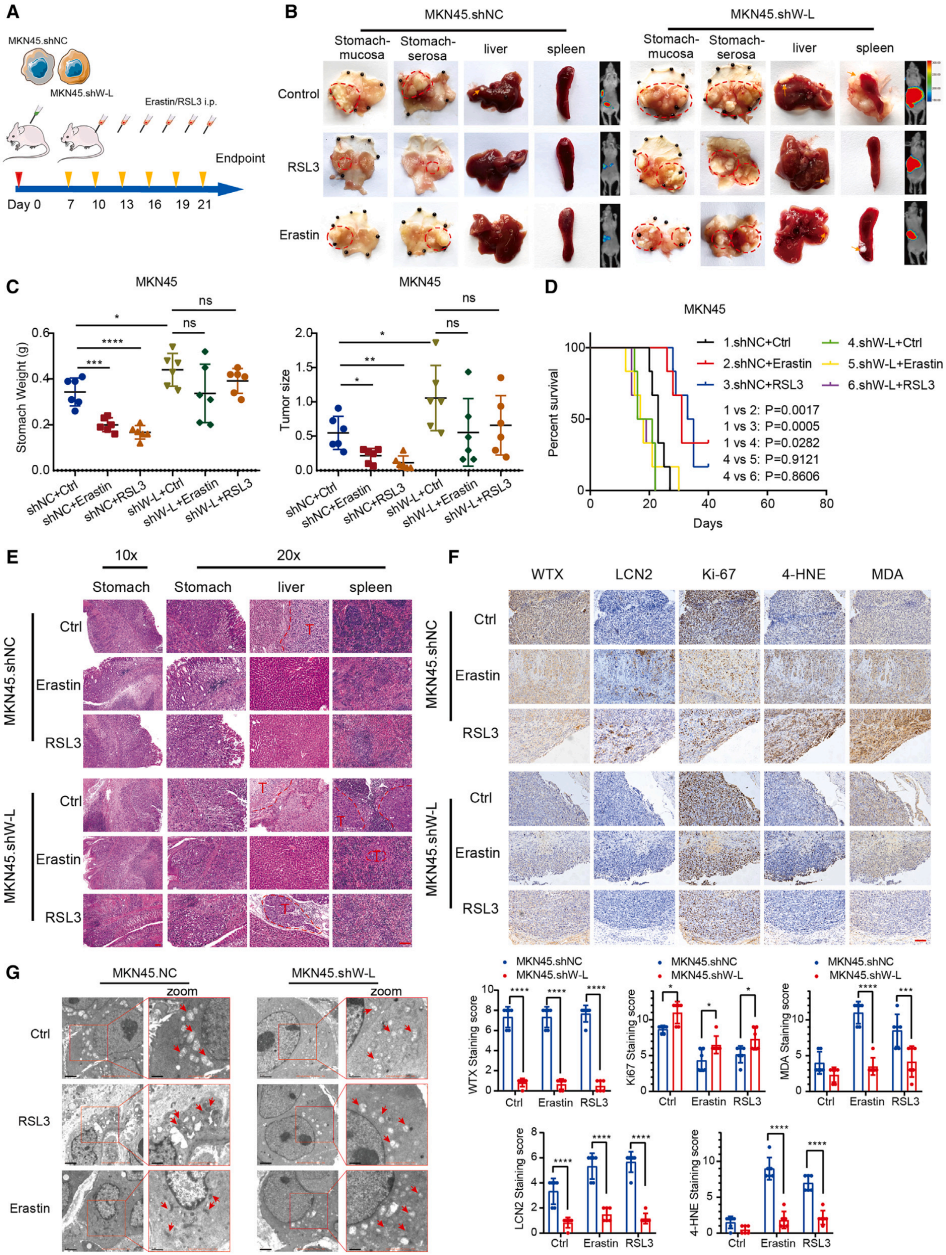
Inhibition of WTX-L impairs sensitivity of GC cells to erastin and RSL3 *in vivo* (A) Schematic diagram showing the erastin and RSL3 treatment processes in MKN45.shNC and MKN45.shW-L cells-derived orthotopic xenograft. (B) Gross GC orthotopic xenografts upon indicated treatment. (C) The tumor weight (left) and volume (right) difference between the indicated groups. (D) Kaplan-Meier survival analysis of nude mice bearing indicated xenografts tumors treated with or without erastin and RSL3. (E) Hematoxylin and eosin staining was used to show the morphological features of stomach, liver, and spleen in the indicated groups of nude mice models. Scale bars, 100 μm. (F) IHC staining reflected expression difference of Ki67, MDA, LCN2, and 4-HNE among the indicated groups. Scale bars, 100 μm. (G) The morphological changes of mitochondria in the indicated groups tumors were detected by transmission electron microscopy. Lower scale bar in zoom, 1 μm. Data are represented as mean ± SEM (n = 3 per group, unless otherwise indicated). ∗*p* < 0.05, ∗∗*p* < 0.01, ∗∗∗*p* < 0.001, ∗∗∗∗*p* < 0.0001, ns: no significance.

To examine the roles of WTX-L inhibition in tumor sensitivity to erastin and RSL3 *in vivo*, we established mouse orthotopic GC models using MKN45.shNC and MKN45.shW-L cells (Figure 5A). Seven days after cells implantation, mice were treated intraperitoneally with erastin (15 mg/kg/day per mouse, twice every other day) or RSL3 (10 mg/kg/day per mouse, twice every other day) or normal saline. Mice with digestive symptoms or moribund appearance were sacrificed at the endpoint, and the gross stomach, liver, and spleen were shown (Figure 5B). As shown in Figure 5C, the stomach weight (left) and tumor size (right) with decreased WTX-L expression were markedly larger than the control group with the same treatment. Consistently, mice in the MKN45.shNC group also showed significantly longer lifespans compared with the MKN45.shW-L group when receiving the same treatment (Figure 5D).

Hematoxylin and eosin (HE) staining of the stomach, liver, and spleen showed that mice in the MKN45.shW-L group had much more liver and spleen metastasis than those in the MKN45.shNC group upon the same treatment (Figures 5E; Table S2). Next, we assessed the levels of WTX-L/LCN2/ferroptosis-related proteins in tumor tissues via immunohistochemistry (IHC) staining of WTX, Ki67, 4-hydroxy-2-noneal (4-HNE), LCN2, and MDA (Figure 5F). Consistent with the *in vitro* results, the staining for LCN2, MDA, and 4-HNE was markedly decreased upon erastin or RSL3 treatment in MKN45.shWTX-L cells, indicating a significant decrease in lipid peroxidation level. Besides, tumor tissues of the MKN45.shWTX-L group also exhibited a higher proliferation level through Ki67 staining. TEM was used to observe the mitochondria morphology in tumor tissues after the indicated treatment (Figure 5G). The results showed that there were many more damaged mitochondria forming double-membrane autophagosomes and eventually fusing with lysosomes in the MKN45.shNC tumors upon treatment with erastin and RSL3. Overall, WTX-L inhibition modulated the anti-tumor activity of ferroptosis-inducing agents in GC *in vivo*.

### Prognostic significance of the WTX-L-LCN2 pathway in human GC

**Figure 6:**
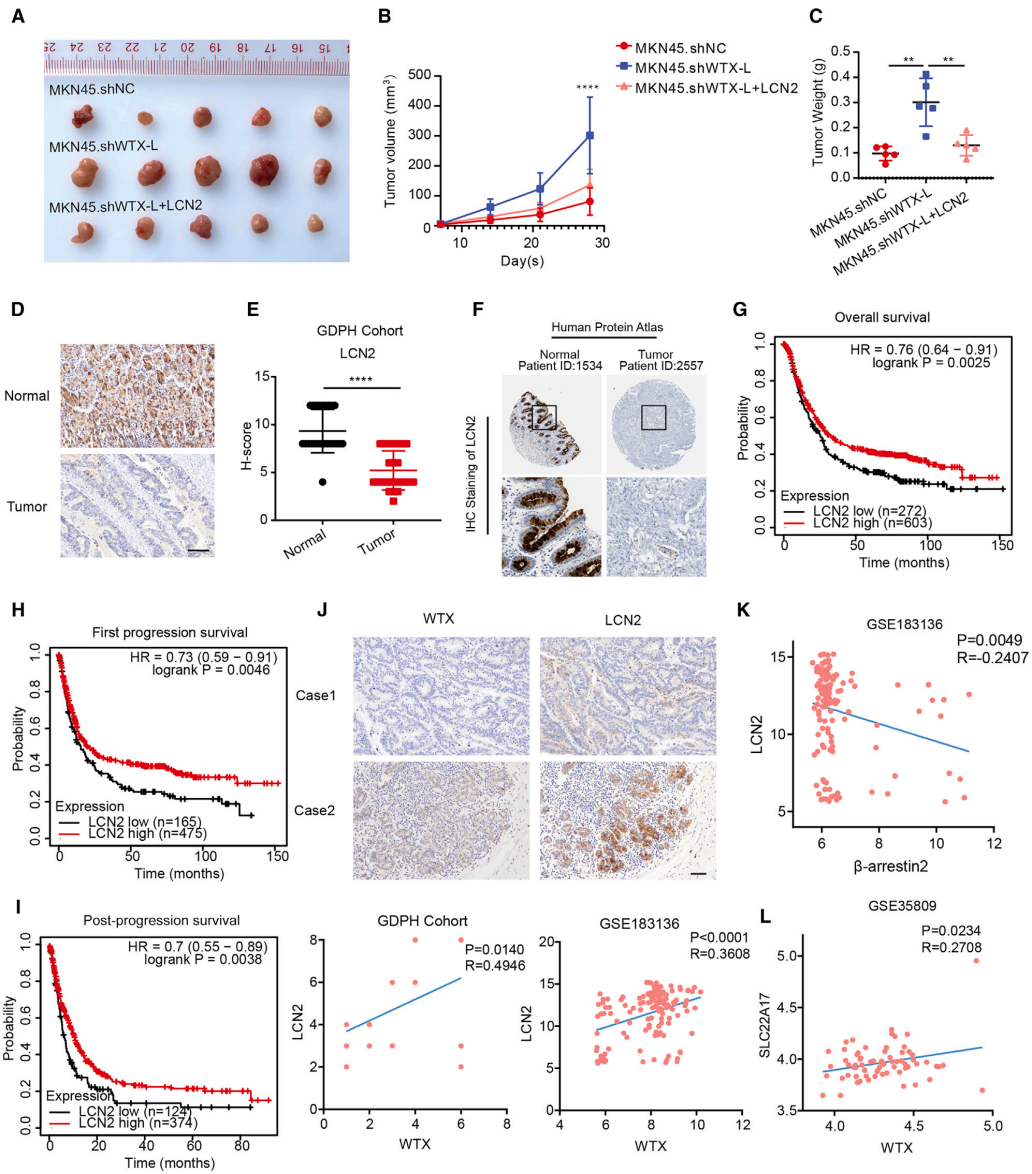
Prognostic significance of the WTX-LCN2 pathway in human GC (A) The image of xenograft tumors developed from the MKN45.shNC, MKN45.shWTX-L, and MKN45.shWTX-L.LCN2 cells. (B) The tumor volumes were measured and calculated at the indicated time (n = 5). (C) The weight of xenograft tumors in MKN45.shNC, MKN45.shWTX-L, and MKN45.shWTX-L.LCN2 groups (n = 5). (D) LCN2 expression was detected by IHC assay in GC and normal gastric tissues from GDPH cohort. Scale bars, 100 μm. (E) Scatterplot showed statistical analysis of the staining intensity score in (D). (F) IHC results of LCN2 protein levels in normal gastric tissue and tumor tissue from the Human Protein Atlas database (HPA: https://www.proteinatlas.org/). (G) Survival curves for patients with GC with low LCN2 expression versus high LCN2 expression from K-M plotter database. (H) First progression survival curves for patients with GC with low LCN2 expression versus high LCN2 expression from K-M plotter database. (I) Post-progression survival curves for patients with GC with low LCN2 expression versus high LCN2 expression from K-M plotter database. (J) LCN2 expression was positively correlated with WTX expression in 24 clinical GC specimens. Shown are visualizations of two representative cases. Bottom: The correlation between LCN2 and WTX was detected in GDPH cohort and GEO: GSE183136 dataset, respectively. Scale bar represents 100 μm. (K) The correlation between β-arrestin2 and LCN2 was detected in GDPH cohort and GEO: GSE183136 dataset. (L) The correlation between WTX and SLC22A17 was detected in GDPH cohort and GEO: GSE35809 dataset. Data are represented as mean ± SEM. ∗∗*p* < 0.01, ∗∗∗∗*p* < 0.0001.

To elucidate the role of LCN2 in WTX-L-mediated GC ferroptosis *in vivo*, we overexpressed LCN2 in MKN45.shWTX-L GC cells and then determined tumor growth. As shown in Figures 6A–6C, restoring LCN2 expression significantly disrupted tumor growth induced by WTX-L knockdown in a subcutaneous tumor model. Finally, we performed IHC and bioinformatics analyses to determine whether the correlation among WTX-L, β-arrestin2, and LCN2 in our GC cell models was also evident in clinical GC specimens. LCN2 protein expression patterns were investigated by IHC staining in 24 pairs of primary GC tissues and matched with adjacent normal tissues from the Guangdong Provincial People’s Hospital (GDPH) cohort, which showed a significantly lower LCN2 expression compared with that matched with normal mucosa (Figures 6D and 6E). LCN2 protein expression was also verified by the Human Protein Atlas (HPA: https://www.proteinatlas.org/) database and consistent result was achieved (Figure 6F). Through analyzing data from K-M plotter database, LCN2 overexpression markedly correlated with better overall survival, first progression, and post-progression survival of patients with GC (Figures 6G–6I). Significant positive correlation between WTX and LCN2 expression was observed in both GDPH cohort and GEO: GSE183136 dataset (Figure 6J). Besides, significant negative correlation between β-arrestin2 and LCN2 expression, as well as positive correlation between WTX and SLC22A17 expression, was also observed in the GEO: GSE183136 (Figure 6K) and GEO: GSE35809 dataset (Figure 6L), respectively. Overall, our findings on the WTX-L/β-arrestin2/LCN2 axis *in vitro* were consistently verified in clinical GC specimens, suggesting that targeting this axis to induce ferroptosis might be a promising option for patients with GC.

## Discussion

Recent studies have proposed the tumor inhibition and metastasis inhibition effects of ferroptosis in GC,^31^ so as to accelerate the clinical application of ferroptosis induction as a therapeutic strategy for GC. However, the precise regulatory networks that underlie ferroptosis are still largely unknown. Although our previous clinicopathological results and biological function experiments suggested that WTX, a tumor suppressor, can be used as a prognosis marker in patients with GC and exhibited its ability to prevent GC cell proliferation, migration, and invasion,^18^^,^^19^ the precise role and molecular mechanisms of WTX to exert its tumor suppression function are still worthy of further investigation. To date, WTX has been reported to be involved in tumor suppression through multiple pathways related to its cellular localization, for example, inhibiting the activity of the WNT signaling pathway by promoting β-catenin degradation in the cytoplasm,^13^ while regulating the transcriptional activity of WT1 in the nucleus.^22^ Although a shorter splice form (WTX-S) appears primarily in the nucleus, cellular localization studies indicate that most nonsplice WTX (WTX-L) resides at the plasma membrane.^32^ The main mechanistic difference between WTX-S and WTX-L is that, in the nucleus, WTX-S colocalizes paraspeckles markers of a nuclear domain associated with transcriptional regulation and, in combination with WT1, is associated with renal and gonadal organogenesis, pleural/pericardial formation, and cardiac stem cell maintenance.^13^ The function of WTX-L is mainly as an inhibitor of Wnt signaling, which is dependent on its membrane localization and cell density,^33^ suggesting that the function of WTX-L membrane localization is worth exploring. In the early stage of our study, we performed immunohistochemical assays to compare the expression levels of WTX-S and WTX-L in 20 patients with GC with high WTX expression. The results indicated that the expression level of WTX-L was significantly higher than that of WTX-S (Figure S4E). In this study, we found that WTX-L, not WTX-S, is the key regulator of ferroptosis. WTX-L localized to the plasma membrane forms a complex with β-arrestin2, disrupting its direct binding to IκIBa. This disruption leads to the upregulation of LCN2 expression, resulting in a significantly increased pool of labile free iron. The excess of free iron triggers an overload of ROS, ultimately inducing ferroptosis. Furthermore, ferroptosis-induced mobility of WTX-L within the membrane enhances its interaction with β-arrestin2, which further promotes LCN2-mediated ferroptosis by inhibiting the phosphorylation of focal adhesion kinase, thereby increasing the susceptibility of cells to ferroptosis. In contrast, WTX-L knockdown protects metastasizing GC cells from ferroptosis *in vivo* and reduces their sensitivity to ferroptosis inducers such as erastin and RSL3. These findings highlight the association between WTX-L expression and the tumor-inhibitory and metastasis-inhibitory effects of ferroptosis in GC.

The mechanism by which WTX-L functions as a key regulator of ferroptosis in GC progression has not been reported previously. Recent study reported that WTX deficiency inhibits ferroptosis in colorectal cancer cell hematogenous metastasis by inhibiting ubiquitination and degradation of SLC7A11 and FTL.^24^ Consistently, we also found the colocalization of WTX-L with SLC7A11 and FTL, suggesting that WTX as a regulator of ferroptosis is prevalent in tumor development. What is different is that we further found that WTX-L, which binds to β-arrestin2, upregulates the expression and extracellular secretion of LCN2. It is known that β-arrestin2 is a scaffold protein that regulates signal transduction by seven transmembrane-spanning receptors^34^ and effectively modulates activation of NF-κB and expression of NF-κB target genes.^28^^,^^35^ As the NF-κB target gene, LCN2 encodes an iron-sequestering factor that binds to specific cell-surface receptors and is transported into cells through receptor-mediated endocytosis. LCN2 is involved in the development of a variety of diseases: non-neoplastic diseases (such as cachexia, pneumonia, and kidney disease^36^^,^^37^) as well as malignant tumors (such as hepatocellular carcinoma and pancreatic cancer^27^^,^^38^). The function of LCN2 depends on whether it is iron bound; that is, iron-loaded LCN2 promotes tumor growth and progression, whereas iron-free LCN2 has anti-tumor activity.^30^^,^^39^ In septic cardiac dysfunction and lung cancer cachexia, exogenous LCN2 induced ferroptosis via increasing the labile iron pool.^40^^,^^41^ In GC, LCN2 inhibits EMT signaling through MMP2 downregulation, resulting in the reduction of proliferation, invasion, and migration of GC cells.^42^ However, the mechanistic details of ferroptosis-related LCN2 signaling in GC are not known. Here, we found that LCN2 upregulation increases the labile iron pool and inhibits focal adhesion kinase phosphorylation, thereby rendering sensitivity to ferroptosis inducers in GC. Since LCN2 is a secreted protein, the possibility exists that iron-loaded LCN2 is rapidly captured by tumor cells due to the enhanced expression of SLC22A17, the LCN2 receptor.^30^ Indeed, SLC22A17 was highly expressed in patients with GC and positively correlated with WTX, thus pointing toward the possibility that the iron-loaded LCN2 transported outside the cell is re-endocytosed. Therefore, further studies assessing the impact of the iron-loaded form of LCN2 on ferroptosis are needed to deepen our understanding of the diverse functions of LCN2.

The GC orthotopic mouse tumor model provides further validation that WTX-L knockdown inhibits ferroptosis, leading to a high rate of liver and spleen metastasis in mice, thereby shortening the survival time of mice. Nevertheless, control mice were more sensitive to ferroptosis inducers, demonstrating that WTX-L-mediated ferroptosis plays an important role in repressing metastasis for GC.

In conclusion, we have elucidated a regulatory mechanism through which WTX-L promotes ferroptosis by modulating the LCN2-mediated labile iron pool. Additionally, we propose a crosstalk mechanism between ferroptosis and tumor suppression, suggesting that WTX-L enhances sensitivity to ferroptosis. Consequently, distinct intervention strategies should be developed for patients with GC based on their levels of WTX-L or WTX-S expression.

### Limitations of the study

This study has certain limitations that warrant careful consideration. The synergistic effect of WTX-L in conjunction with ferroptosis inhibitors requires further validation in models that more accurately reflect the biological characteristics of GC, such as patient-derived xenografts (PDX) and organoid models. Additionally, the WTX-L protein has a large structure and a molecular weight exceeding 100 kDa, making it challenging to purify recombinant proteins with *in vivo* anticancer activity directly. Consequently, developing drugs based on WTX-L presents a topic deserving of in-depth exploration. By conducting a thorough analysis of the WTX-L protein structure, constructing truncated carriers to identify the key domain responsible for WTX-L-mediated GC ferroptosis, and designing active peptides based on this domain, we anticipate facilitating the clinical application of this study, which will be a focal point of our future research.

## Resource availability

### Lead contact

Further information and requests for resources and reagents should be directed to and will be fulfilled by the lead contact, Qingling Zhang (zhangqingling@gdph.org.cn).

### Materials availability

This study did not generate new unique reagents.

### Data and code availability

• Three GC datasets analyzed in the current study were downloaded from GEO datasets (GSE62254, GSE183136, and GSE35809). Correlation between survival time and gene expression levels was analyzed in the K-M plotter database. The RNA-seq data generated in this study are available in SRA database, and the accession number is PRJNA1194795.
• This article does not report any original code.
• Any additional information required to reanalyze the data reported in this paper is available from the lead contact upon request.

## Acknowledgments

This project was supported by grants from 10.13039/501100015956Key Area Research and Development Program of Guangdong Province (2021B0101420005), 10.13039/501100001809National Natural Science Foundation of China (Q.Z., 82173033; Y.X., 82102712), 10.13039/501100002858China Postdoctoral Science Foundation (Y.X., 2021M690751), 10.13039/501100018609High-level Hospital Construction Project (Q.Z., DFJHBF202108), and Guangdong Provincial Key Laboratory of Artificial Intelligence in Medical Image Analysis and Application (No. 2022B1212010011).

## Author contributions

Y.X.: project administration, data curation, validation, writing – original draft, writing – review & editing, funding acquisition. X.Q.: investigation, formal analysis, data curation, methodology. G.C.: validation, project administration, methodology. Z.L.: duplication validation, project administration, methodology. W.H.: project administration, methodology. C.W.: supervision, resources. Y.Z. and J.S.: visualization, validation. H.W.: visualization, supervision. Q.Z.: writing – review & editing, conceptualization, supervision, funding acquisition. All authors have read and agreed to the published version of the manuscript.

## Declaration of interests

The authors declare no competing interests.

## STAR★Methods

### Key resources table

**Table:** 

REAGENT or RESOURCE	SOURCE	IDENTIFIER
**Antibodies**		

GFP	Proteintech	Cat#66002-1-Ig; RRID: AB_11182611
LCN2	Abcam	Cat#ab63929; RRID: AB_1140965
β-arrestin2	Cell Signaling Technology	Cat#4674S
WTX-WT	This paper	N/A
WTX-L	This paper	N/A
WTX-S	This paper	N/A
P65	Cell Signaling Technology	Cat#8242; RRID: AB_2860400
p-p65	Cell Signaling Technology	Cat#3033; RRID: AB_895741
FAK	Proteintch	Cat#12636-1-AP; RRID: AB_2173668
*p*-FAK	Proteintch	Cat#83933-1-RR; RRID: AB_10780397
GAPDH	Proteintch	Cat#60004-1-Ig; RRID: AB_2107436
β-Tubulin	Cell Signaling Technology	Cat#2146; RRID: AB_2256738
FTL	Proteintch	Cat#10727-1-AP; RRID: AB_2278673
SLC7A11	HUABIO	Cat#HA600098; RRID: AB_3071714

**Biological samples**		

44 cases of GC and matched adjacent normal gastric tissue samples	Guangdong Provincial People’s Hospital	N/A

**Chemicals, peptides, and recombinant proteins**		

Erastin	Selleck Chemicals	Cat# S7242
ferrostatin-1	Selleck Chemicals	Cat# S7243
RSL3	Selleck Chemicals	Cat# S8155
DFO	Selleck Chemicals	Cat# S5742
NAC	Selleck Chemicals	Cat# S5804
Z-VAD-FMK	Selleck Chemicals	Cat# S7023
necrosulfonamide	Selleck Chemicals	Cat# S8251
Lipofectamine 2000	Invitrogen	Cat# 11668019
siRNA Transfection Reagent	Beyotime	Cat# C0526
Trizol reagent	Clontech Laboratories	N/A

**Critical commercial assays**		

Cell Counting Kit-8	DOJINDO	Cat#CK04
PrimeScript™ RT reagent Kit	Clontech Laboratories	N/A
SYBR Green™ Fast qPCR Mix	Takara	N/A
mycoplasma contamination test kit	Servicebio	Cat#G1901-20T
Cell Ferrous Iron Colorimetric Assay Kit (4-HNE) Assay Kit	Elabscience	Cat#E-BC-K881-M
Lipid Peroxidation (MDA) Assay Kit	Sigma-Aldrich	Cat#MAK085

**Deposited data**		

RNA-seq (PRJNA1194795)	This paper	https://www.ncbi.nlm.nih.gov/sra
GSE62254	GEO	https://www.ncbi.nlm.nih.gov/geo/
GSE183136	GEO	https://www.ncbi.nlm.nih.gov/geo/
GSE35809	GEO	https://www.ncbi.nlm.nih.gov/geo/
Survival data of GC patients	K-M Plotter	https://kmplot.com/analysis/index.php?p=service&cancer=gastric

**Experimental models: Cell lines**		

Human: AGS cells	ATCC	Cat#CRL-1739
Human: MKN-45 cells	Cellbank, RIKEN BRC	RCB1001
Human: GES-1 cells	Biofeng	N/A
Human: KATO-III cells	ATCC	Cat# HTB-103
Human: NCI-N87 cells	ATCC	Cat# CRL-5822

**Experimental models: Organisms/strains**		

BALB/c nude mice (nu/nu)	Guangdong Medical Laboratory Animal Center	N/A

**Oligonucleotides**		

Sequences for siRNAs, see Table S5	This paper	N/A
Primers for RT-PCR, see Table S6	This paper	N/A

**Software and algorithms**		

GraphPad Prism 8	GraphPad Software Inc.	http://www.graphpad.com
Gene Set Enrichment Analysis (GSEA)	Broad Institute	http://software.broadinstitute.org/gsea/index.jsp
ImageJ	National Institutes of Health	https://imagej.nih.gov/ij
FlowJo 10.8.1	FlowJo Software	https://www.flowjo.com/
Adobe Illustrator	Adobe	https://www.adobe.com/products/illustrator.html

### Experimental model and study participant details

#### Clinical samples

44 cases of GC and matched adjacent normal gastric tissue samples were obtained from patients at Guangdong Provincial People’s Hospital (GDPH), Southern Medical University. The adjacent normal gastric samples were allocated to the control groups. Prior patient consent and approval were obtained from the Institutional Research Ethics Committee (Study approval number: KY-Z-2021-493-01). The clinical and demographic information of the patient has been provided in Tables S3 and S4.

#### Cell culture

Human GC cell lines, AGS, NCI-N87, and KATO-III, were purchased from the American Type Culture Collection (ATCC, USA), generated, in the Department of Pathology, Nanfang Hospital. MKN45 cells were obtained from the cell bank of the RIKEN BioResource Center (Tsukuba, Japan).

All human gastric cells were cultured using RPMI-1640 medium (Gibco, USA) supplemented with 10% FBS (Gibco, USA), 100 U/mL penicillin and 0.1 g/mL streptomycin (Sigma, USA) in a humidified 37°C incubator with 5% CO_2_. All cell lines were routinely tested for mycoplasma, the results of which were negative.

#### Animal experiments

Four-to six-week-old male BALB/c nude mice were purchased from Guangdong Medical Laboratory Animal Center (Guangdong, China) and treated according to the Animal Research Reporting of *In vivo* Experiments (ARRIVE) guidelines. All animal studies were approved by the Animal Care and Use Committee of Guangdong Provincial People’s Hospital, Southern Medical University (Study approval number: KY-D-2021-126-01).

For the subcutaneous tumor model, 2 × 10^6^ GC cells were suspended in 100 μL of PBS and injected subcutaneously into the right inguinal region of each nude mouse. The tumor volumes were measured every 7 days starting 7 days after injection. After 28 days, mice were sacrificed, and the tumors were collected for further analysis and weighed.

For the orthotopic GC mouse models, the mice were anesthetized with ketamine (70 μg/kg), followed by a small abdominal incision to expose the stomach. A total of 100 μL of a cell suspension (1 × 10^6^ cells) was injected into the muscle tissue of the stomach. After resetting the stomach position, the wound was treated with penicillin, and the abdominal incision was closed. One week later, the mice were randomly allocated into groups and treated with either erastin (15 mg/kg/day per mouse, twice every other day), RSL3 (10 mg/kg/day per mouse, twice every other day), or normal saline via intraperitoneal injection for 20 days. For the dissolution methods of erastin and RSL3, please refer to the Selleck websites (https://www.selleck.cn/products/erastin.html and https://www.selleck.cn/products/rsl3.html, respectively). To enhance the solubility of erastin, the tube was warmed in a 37°C water bath and gently shaken. All animal procedures were conducted in accordance with institutional guidelines. Mice exhibiting digestive symptoms or signs of poor health were euthanized. At the conclusion of the experiment, all mice were sacrificed, and the tumors were harvested and weighed.

Tumor volumes were calculated using the formula: length × (width)^2/2^. A portion of each tumor tissue was fixed in 4% paraformaldehyde and embedded in paraffin for IHC and HE analysis.

### Method details

#### Overexpression and knockdown cell lines

A lentiviral expression vector (LV-WTX-WT-Puro) containing the complete WTX coding sequence (CDS) was synthesized by Genechem Co. (Shanghai, China). The WTX-S (del50-326aa) and WTX-L variants were generated by modifying the WTX-WT vector through PCR cloning. WTX-L incorporates a single nucleotide change that disrupts the internal splice acceptor site (150 T>C). WTX shRNA fragments were synthesized by Tsingke Biotech Co. (Beijing, China), verified, and subsequently utilized to establish a lentiviral expression vector (LV-shWTX-L-Puro). Target cells (2 × 10^5^) were infected with 1 × 10^6^ lentiviral transducing units in the presence of polybrene (1 μg/mL) and were then selected with puromycin (2 μg/mL) for approximately 5 days to establish stable overexpression and knockdown cell lines. Plasmids were transferred to GC cell lines using lipofectamine 2000 (Invitrogen, 11668019). SiRNAs targeting LCN2 and β-arrestin2 were transfected with siRNA Transfection Reagent (Beyotime, C0526). The specific sequence information for siRNA is presented in Table S5.

#### Cell viability assay

The cell viability was measured in different groups using Cell Counting Kit-8 (CCK-8, Dojindo, Japan) as the Feng et al.^43^ In brief, 10μL of the CCK-8 reagent was added to each well (containing 100μL of medium) on the 96-well microplate, and the microplate was incubated at 37°C for 2h. Finally, the OD (450nm) was detected in each group (*n* = 3). The cell viability in the Ctrl group (without any treatment) was regarded as “100%”, and the relative cell viability of the other groups was evaluated respectively.

#### Quantitative reverse transcription PCR (RT-qPCR) and RNA sequencing

Real-time qPCR was then performed as our previously described.^19^ Briefly, total RNA was extracted from tissue and cells using TRIzol reagent (Clontech Laboratories) according to the manufacturer’s instructions. Reverse transcription was performed using PrimeScript RT Master Mix (Clontech Laboratories). Quantitative real-time PCR analysis was performed using SYBR Green PCR Master Mix (TaKaRa). The sequences of all indicated primers are listed in Table S6. For RNA sequencing, the enriched mRNA from AGS.Vector and AGS.WTX-L cells treated with or without Erastin (6 μM)/RSL3 (3 μM) was reverse transcribed into cDNA. The cDNA libraries were sequenced on the BGI sequencing platform by Benagen Biotechnology Co., Ltd (Wuhan, China).

#### Immunoprecipitation and immunoblotting

The proteins were eluted with denaturation buffer, separated by SDS-PAGE, and transferred to PVDF membranes (Pierce Biotechnology, Rockford, IL, USA). Subsequently, the membranes were blocked with milk before being incubated with primary antibodies. For immunoprecipitation, cellular lysates were incubated with antibodies overnight, followed by a 4-h incubation with protein A/G-sepharose beads (Santa Cruz Biotechnology). This was followed by centrifugation at 2500 × g and washing three times with ice-cold lysis buffer. The immunoprecipitated proteins were eluted with denaturation Laemmli buffer at 95°C for 5 min, separated by SDS-PAGE, transferred to a PVDF membrane, blocked with 5% nonfat milk, and then incubated with primary and secondary antibodies. The proteins were finally visualized using an enhanced chemiluminescence detection system (Amersham Biosciences Europe, Freiberg, Germany) according to the manufacturer’s instructions.

#### Fluorescence Recovery after photo bleaching (FRAP)

FRAP assay was conducted using the FRAP module of the Carl Zeiss (LSM 900 Meta plus Zeiss Axiovert zoom) confocal fluorescence microscopy system. Defined regions were photobleached at 488 nm. The fluorescence intensities in these regions were collected every 2 or 3 s and normalized to the initial intensity before bleaching. Image intensity was measured by Mean ROI and further analyzed by GraphPad Prism 8.0.

#### Immunofluorescence (IF) of cell staining

Transfected cells were fixed with 4% paraformaldehyde (Biosharp) in PBS for 10 min, followed by permeabilization with 0.2% Triton X-100 in PBS for 10 min, and then blocked with 1% BSA for 1h, with all steps performed in room temperature. Samples were further incubated with primary antibodies in blocking buffer overnight at 4°C, then washed 3 times with PBST (0.1% Tween) followed by incubation with secondary antibody for 1h at room temperature. Host-specific Alexa Fluor 488/594/647 (Proteintech) secondary antibodies were used for visualization. For microscopic imaging, slides were mounted with DAPI (Solarbio). Images were captured using a Carl Zeiss Confocal fluorescence microscope with a 63× oil objective. Images were post-processed using Imaris v9.0.0 Bitplane Inc. The following primary antibodies were used: FTL (Proteintech, 10727-1-AP, 1:200 dilution), SLC7A11 (HUABIO, HA600098, 1:200 dilution).

#### Evaluation of malondialdehyde (MDA) and iron level

In this study, the MDA and Fe^2+^ in different groups were measured to evaluate ferroptosis level. The MDA concentration and Fe^2+^ concentration in cell lysates were assessed using the Lipid Peroxidation (MDA) Assay Kit (MAK085, Sigma-Aldrich, USA), and Cell Ferrous Iron Colorimetric Assay Kit (4-HNE) Assay Kit (E-BC-K881-M, Elabscience) according to the manufacturer’s instructions.

#### Fluorescent probes staining

An equal amount of cells were treated as designed and stained with the fluorescent probe of DCF-DA (5μM), BODIPY (4μM), and MitoSOX (3μM), which were used for detecting the level of cellular ROS, lipid peroxides, and mitochondrial ROS, respectively. After incubation for 30 min at 37°C, cells were washed three times with HBSS and subsequently detected by flow cytometry or confocal microscopy.

#### Collection of secreted proteins mixture

The collection of secreted protein mixtures was conducted as previously described.^44^ Briefly, prior to harvesting the secreted LCN2 proteins, cells were cultured under serum starvation conditions for 24 h. The media were collected and filtered through a 0.22 μm filter (Millipore) to eliminate any dead cells. The samples were subsequently transferred to an Amicon Ultra-4 10K molecular weight cut-off centrifugal filter (Millipore) and centrifuged until the remaining volume reached the minimum. The concentrated secreted protein mixture was then transferred to new tubes.

#### Transmission electron microscope (TEM)

After the indicated treatments, the cells were fixed in a 2.5% glutaraldehyde solution at 4°C overnight. Subsequently, the samples were dehydrated using a graded series of ethanol and then transferred to absolute acetone. Following infiltration with absolute acetone and the final Spurr resin mixture, the samples were embedded, ultrathin sectioned, and stained. Finally, the samples were observed using a Hitachi Model H-7650 TEM.

#### Immunohistochemistry (IHC)

Human and mice GC tissues (0.5–1 cm^3^) were fixed in 10% neutral buffered formalin, dehydrated, and embedded in paraffin. Tissue sections of 2.5μm thickness were incubated in citrate buffer (pH 6.0) for 5 min at 120°C, and the endogenous peroxidase was blocked by 0.3% H_2_O_2_ for 10 min. The tissue was then incubated with 5% BSA in PBS for 30 min at 37°C to block the non-specific binding sites, followed by incubation with appropriate primary antibodies overnight at 4°C, and then with horseradish peroxidase (HRP) anti-rabbit IgG or anti-mouse IgG antibodies for 1 h. The color was then developed by incubation with DAB Substrate kit (Zsbio, ZLI-9017). After washing in PBS, tissue sections were counterstained with hematoxylin and examined under a microscope.

### Quantification and statistical analysis

All experiments were conducted independently three times with consistent results. Data are presented as the mean ± SD, and statistical significance was determined by Student’s t test. All statistical analyses were performed using GraphPad Prism 8 software. A *p* value <0.05 (*p* < 0.05) was considered to indicate statistical significance.

## Supplemental information

Document S1. Figures S1–S4 and Tables S2–S6

Table S1. GO analysis of differentially expressed genes between AGS.Vector and AGS.WTX-S cells
