# Supplementary material for: WTX-L/β-arrestin2/LCN2 axis controls vulnerability to ferroptosis in gastric cancer
Source: iScience. 2025 Feb 7;28(3):111964. doi: 10.1016/j.isci.2025.111964 (PMC11919608; doi:10.1016/j.isci.2025.111964)
Supplement: Document S1. Figures S1–S4 and Tables S2–S6 [file mmc1.pdf]

iScience, Volume *28*

Supplemental information

WTX-L/β-arrestin2/LCN2 axis controls vulnerability to ferroptosis in gastric cancer

Yangwei Xu, Xuexia Qian, Guixing Cai, Zhihao Lin, Weiye Huang, Chuangyuan Wang, Hongmei Wu, Yiqiong Zhang, Jingbo Sun, and Qingling Zhang

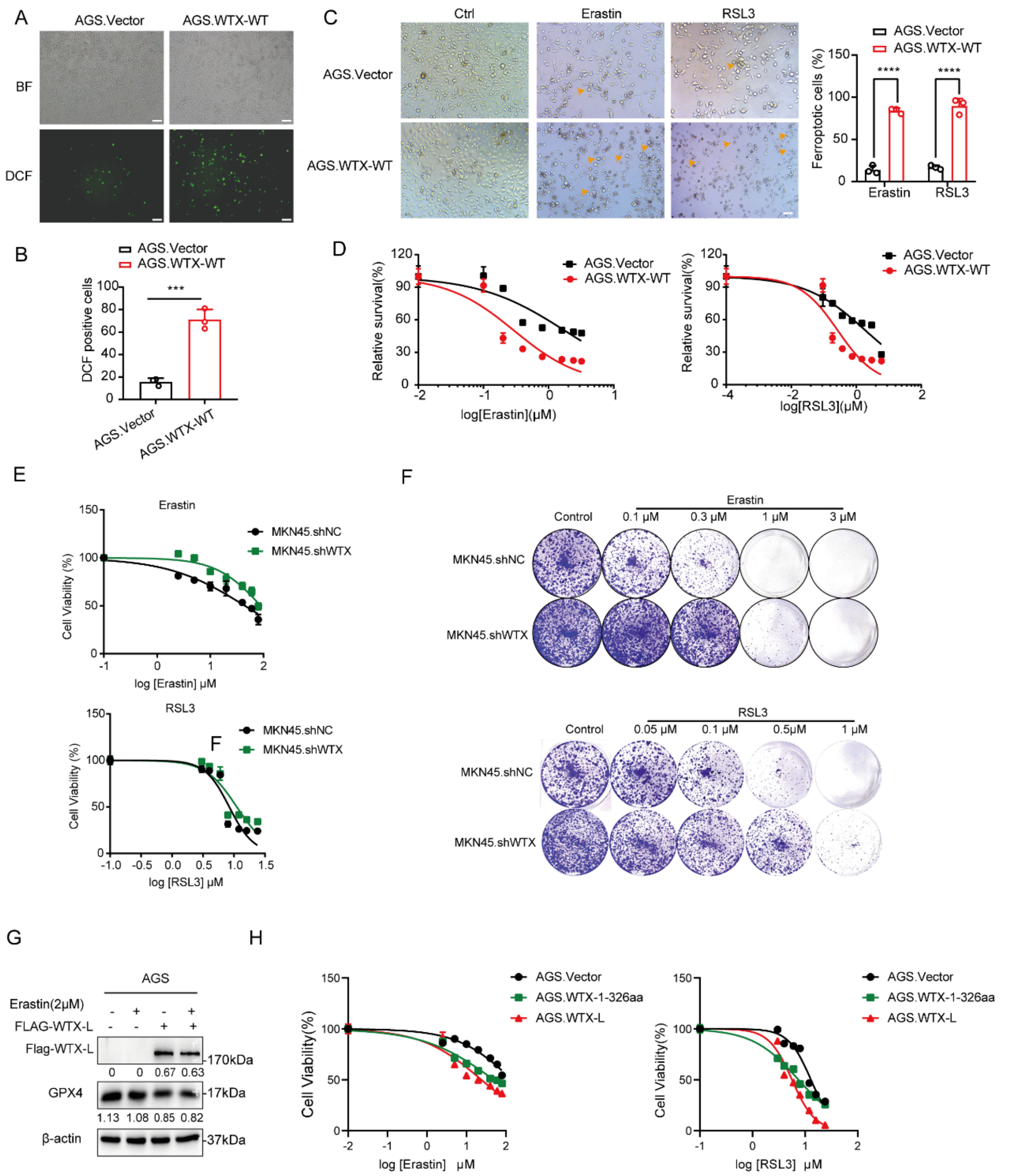

Figure S1. WTX acts as a ferroptosis regulator.

(A) The AGS.Vector and AGS.WTX-WT cells were treated with 3 μM DCF for 24 h. Immunofluorescence assay was performed to determine the level of lipid ROS. Scale bars: 50 μm.

(B) Percentage of DCF positive cells from (A).

(C) Cell morphology of AGS.Vector and AGS.WTX-WT exposed to erastin and RSL3 was monitored by using the Olympus imaging system. Scale bars: 20 μm.

(D) The AGS.Vector and AGS.WTX-WT were exposed to indicated concentrations of erastin and RSL3 for 24h. Cell viability was measured by CCK8 assay.

(E) The MKN45.shNC and MKN45.shWTX cells were exposed to indicated concentrations of

erastin and RSL3 for 24h. Cell viability was measured by CCK8 assay.

(F) Colony formation assay analysed the cell survival of MKN45.shNC and MKN45.shWTX cells exposed to indicated concentrations of erastin and RSL3.

(G) Western blot showed the GPX4 protein expression levels of indicated AGS cells.

(H) The AGS.Vector, AGS.WTX-1-326aa and AGS.WTX-L cells were exposed to indicated concentrations of erastin and RSL3 for 24h. Cell viability was measured by CCK8 assay.

**$p < 0.01$, ***$p < 0.001$.

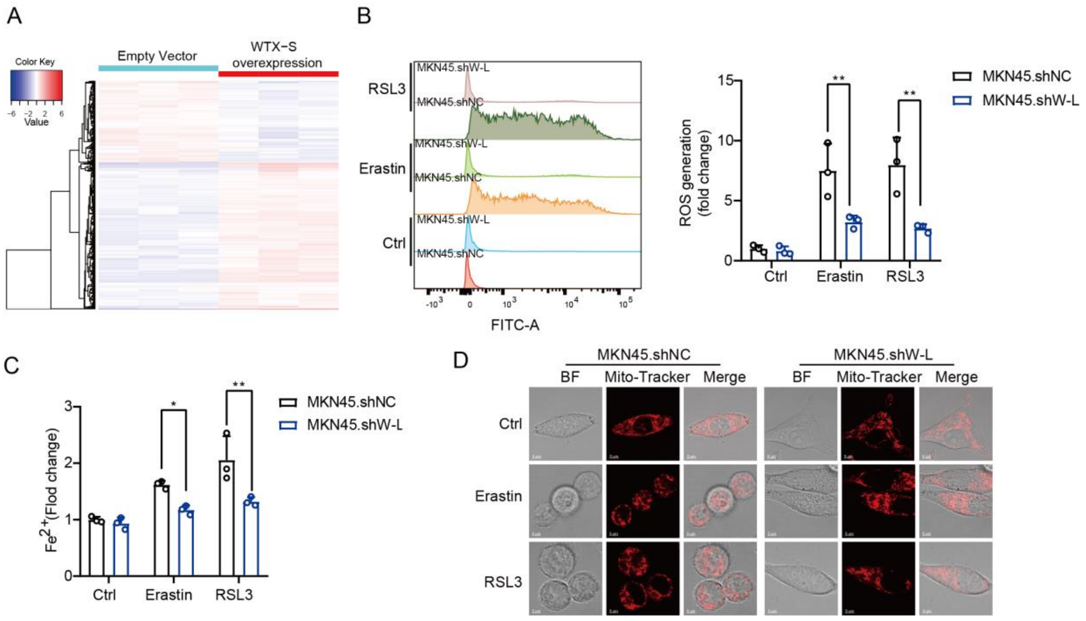

Figure S2. WTX-L promotes iron-dependent oxidative damage in ferroptosis.

(A) Hierarchical clustering analysis of differentially expressed genes between the AGS.WTX-S and AGS.Vector cells (fold change＞2.0).

(B) Flow cytometry assay detected intracellular ROS levels in MKN45.shNC and MKN45.shW-L cells exposed to 3μM erastin and RSL3 for 8h.

(C) Intracellular Fe 2+ levels in in MKN45.shNC and MKN45.shW-L cells following treatment with erastin or RSL3 for 24h were detected through iron detection assay.

(D) Confocal images showed the mitochondria morphology in MKN45.shNC and MKN45.shW-L cells stained with Mito-Tracker. Scale bars: 5 μm.

*$p < 0.05$, **$p < 0.01$.

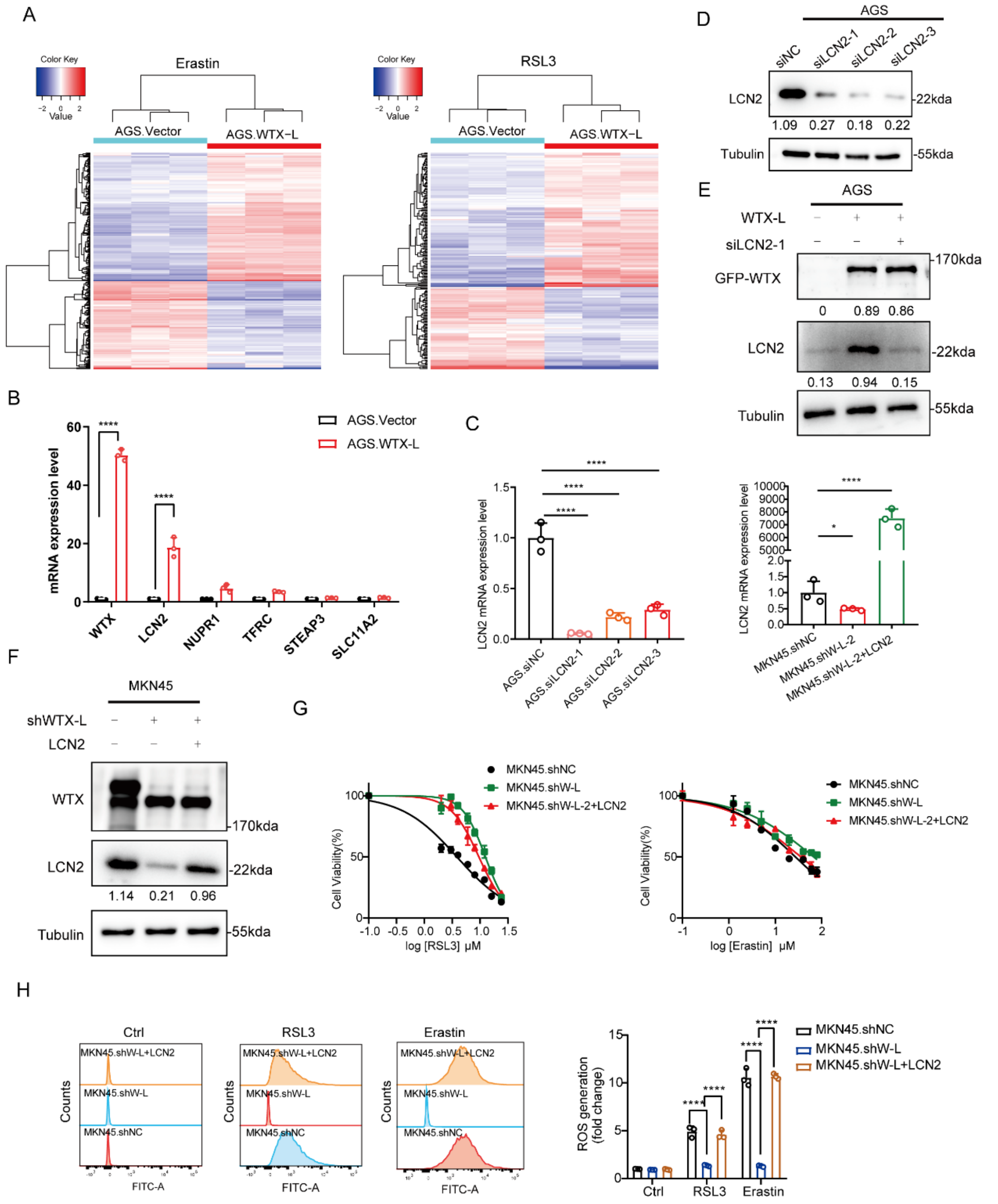

Figure S3. LCN2 acts as an effector gene in WTX-L-mediated ferroptosis sensitivity.

(A) Hierarchical clustering analysis of differentially expressed genes between the AGS.WTX-L and AGS.Vector cells treated with 3μM erastin or 1μM RSL3 for 24h. (fold change＞2.0).

(B) RT-qPCR showed the indicated mRNA levels of AGS.Vector and AGS.WTX-L cells.

(C) RT-qPCR showed the LCN2 mRNA levels of indicated AGS and MKN45 cells.

(D) Western blot showed the LCN2 protein expression levels of AGS cells by using indicated siRNA .

(E) Western blot showed the LCN2 protein expression levels in indicated AGS cells.

(F) Western blot showed the LCN2 protein expression levels in indicated MKN45 cells.

(G) MKN45 cells of indicated groups were exposed to multiple concentrations of Erastin (left panel) and RSL3 (right panel) for 24 h. The cell viability was determined by CCK8 assays.

(H) Flow cytometry assay detected the intracellular ROS levels in indicated MKN45 cells following treatment with 3μM erastin or 1μM RSL3 for 24h.

$^*p < 0.05$, $^{****}p < 0.0001$.

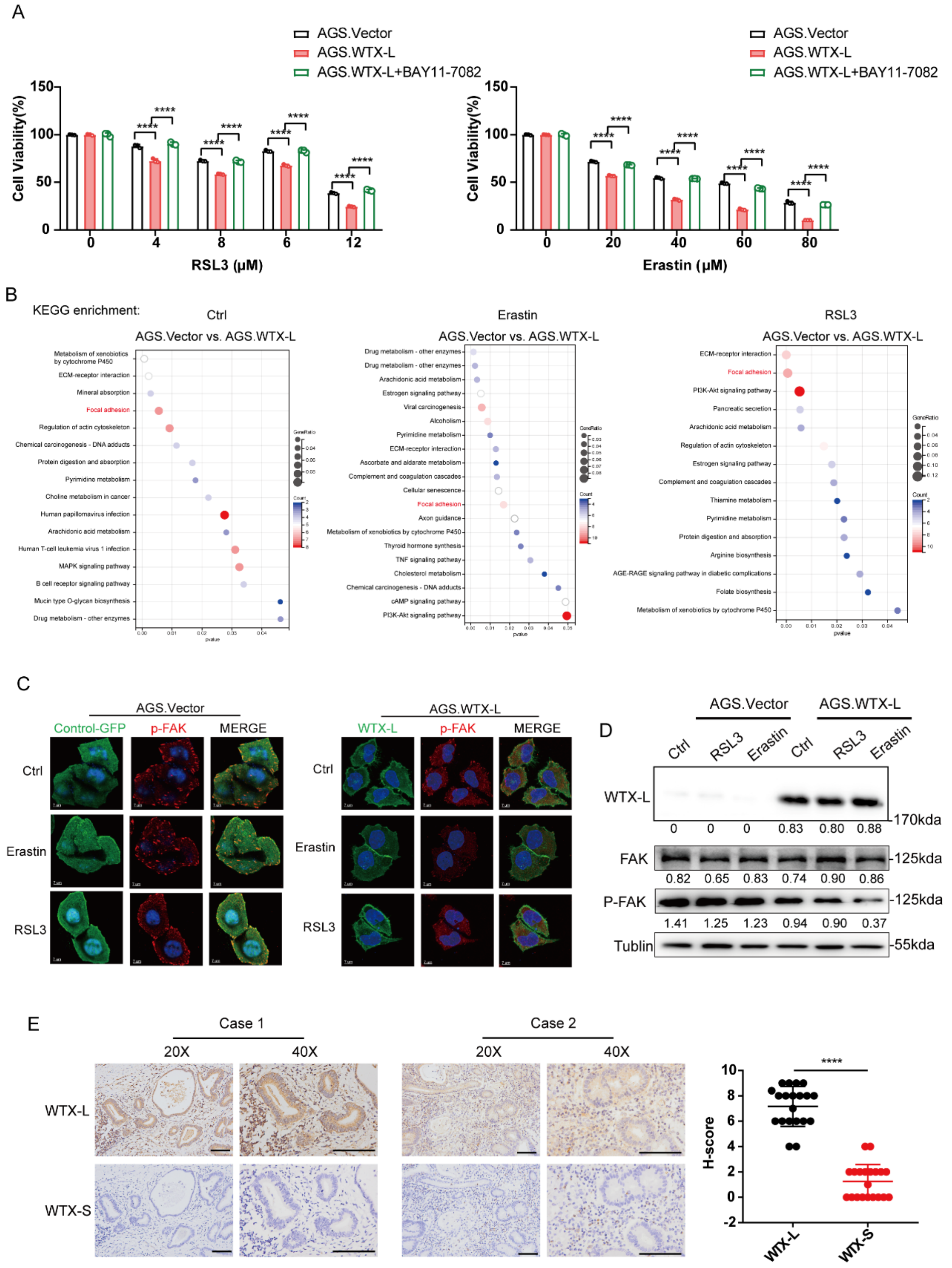

Figure S4. WTX-L-mediated LCN2 inhibits the focal adhesion pathway.

(A) AGS.Vector and AGS.WTX-L cells were exposed to indicated concentrations of erastin (above panel) and RSL3 (below panel) for 24h by using BAY11-7082 (12.5μM). Cell viability was measured by CCK8 assay.

(B) KEGG analysis of differentially expressed genes between the AGS.Vector and AGS.WTX-L

cells with or without 3μM erastin or 1μM RSL3(fold change＞2.0).

(C) Confocal images of p-FAK (red) and WTX-L (green) in AGS.Vector (left panel) and AGS.WTX-L (right panel) cells after indicated treatment. Scale bars: 7 μm.

(D) Western blot showed the expression levels of FAK, P-FAK, and GFP-WTX in AGS.Vector and AGS.WTX-L cells after indicated treatment.

(E) IHC staining and reflected expression difference of WTX-L and WTX-S in high WTX-WT expression of GC groups. Scale bars: 100 μm

Supplementary Table S2: WTX modified orthotopic xenograft GC tumor formation and liver/spleen metastasis analysis.

	Total mice number	Orthotropic tumor N (%)	High tumor burden N (%)	Liver metastasis N (%)	Spleen metastasis N (%)
MKN45.shCtrl	6	6 (100)	6 (100)	3 (50)	4 (66.67)
MKN45.shCtrl+ Erastin	6	6 (100)	2 (33.33)	2 (33.33)	2 (33.33)
MKN45.shCtrl+ RSL3	6	6 (100)	0 (0)	1 (16.67)	2 (33.33)
MKN45.shWTX	6	6 (100)	6 (100)	4 (66.67)	5 (83.33)
MKN45.shWTX +Erastin	6	6 (100)	5 (83.33)	3 (50)	2 (33.33)
MKN45.shWTX +RSL3	6	6 (100)	5 (83.33)	3 (50)	3 (50)

High tumor burden: The tumor mass is more than 1cm in diameter

Table. S3 Clinical and demographic data of the collector of paraffin-fixed samples, related to Figure 6D.

Number	Gender	Age	Race	Tumor Diameter （cm）	T classification	N stage	M stage
1	Male	50	Asian (China)	4.8	3	0	0
2	Male	59	Asian (China)	2.8	3	3	0
3	Male	70	Asian (China)	3.5	3	1	0
4	Male	63	Asian (China)	4.5	3	0	0
5	Female	38	Asian (China)	7.5	2	3	1
6	Male	71	Asian (China)	7.0	3	1	0
7	Female	60	Asian (China)	7.0	4	1	1
8	Male	73	Asian (China)	0.3	2	1	0
9	Female	67	Asian (China)	1.5	2	1	0
10	Female	51	Asian (China)	4.5	2	1	0
11	Female	45	Asian (China)	4.4	3	3	0
12	Male	66	Asian (China)	1.5	3	0	0
13	Female	56	Asian (China)	4.0	2	3	0
14	Female	68	Asian (China)	1.0	3	0	0
15	Male	63	Asian (China)	4.5	3	0	0
16	Male	61	Asian (China)	1.0	2	0	0
17	Female	62	Asian (China)	4.5	1	0	0
18	Male	62	Asian (China)	5.1	3	0	0
19	Male	57	Asian (China)	6.3	3	0	0
20	Male	60	Asian (China)	5.0	3	3	1

21	Male	63	Asian (China)	5.5	3	1	0
22	Male	45	Asian (China)	4.5	1	0	0
23	Male	63	Asian (China)	1.5	3	0	0
24	Female	52	Asian (China)	4.0	3	1	0

Table. S4 Clinical and demographic data of the collector of paraffin-fixed samples, related to Figure S4E.

Number	Gender	Age	Race	Tumor Diameter（cm）	T classification	N stage	M stage
1	Male	56	Asian (China)	7.0	2	3	0
2	Male	69	Asian (China)	3.0	3	2	0
3	Male	65	Asian (China)	5.0	3	1	0
4	Male	58	Asian (China)	3.5	1	0	0
5	Male	55	Asian (China)	8.0	3	0	0
6	Male	68	Asian (China)	2.6	3	0	0
7	Male	70	Asian (China)	4.0	3	1	0
8	Male	67	Asian (China)	5.0	3	2	0
9	Female	67	Asian (China)	3.0	3	0	0
10	Female	51	Asian (China)	3.0	3	1	0
11	Male	46	Asian (China)	6.0	3	3	0
12	Male	63	Asian (China)	3.8	4	0	0
13	Male	55	Asian (China)	10.5	4	3	1
14	Male	56	Asian (China)	7.5	3	1	0
15	Male	69	Asian (China)	2.0	4	0	0
16	Male	58	Asian (China)	4.0	4	3	0
17	Male	45	Asian (China)	3.0	3	1	0
18	Male	63	Asian (China)	3.8	4	2	0
19	Male	78	Asian (China)	6.6	3	2	0
20	Male	62	Asian (China)	8.0	4	3	0

Supplementary Table S5. siRNA sequences

Gene name	Target sequance	sense（5'-3'）	antisense（5'-3'）
siLCN2-1	GAACTTCCAGGACAACCAA	GAACUUCCAGGACAACCAA(dT)(dT)	UUGGUUGUCCUGGAAGUUC(dT)(dT)
siLCN2-2	CCACCATCTATGAGCTGAA	CCACCAUCUAUGAGCUGAA(dT)(dT)	UUCAGCUCAUAGAUGGUGG(dT)(dT)
siLCN2-3	GGAGCTGACTTCGGAACTA	GGAGCUGACUUCGGAACUA(dT)(dT)	UAGUUCCGAAGUCAGCUCC(dT)(dT)

siARRB2-1	CGUAGAUCACCUGGACAAA	CGUAGAUCACCUGGA CAAA(dT)(dT)	UUUGUCCAGGUGA UCUACG(dT)(dT)
siARRB2-2	CGAACAAGAUGACCAGGUA	CGAACAAGAUGACCA GGUA(dT)(dT)	UACCUGGUCAUCUU GUUCG(dT)(dT)

Supplementary Table S6. RT-qPCR primer sequences for human genes

**Gene**	**Forward primer**	**Reverse primer**	**Product length**
GAPDH	GGAGCGAGATCCCTCCAAAAT	GGCTGTTGTCATACTTCTCATGG	197bp
LCN2	GACAACCAATTCCAGGGGAAG	GCATACATCTTTTGCGGGTCT	89bp
WTX	GGCAGCTCCAAGAAAGGTCT	GCTCCAGCCACAGATGTCTT	200bp
ABBR2	TCCATGCTCCGTCACACTG	ACAGAAGGCTCGAATCTCAAAG	82bp
